# The changing profile of the internationally educated nurse workforce: Post-pandemic implications for health human resource planning

**DOI:** 10.1177/08404704231198026

**Published:** 2023-08-31

**Authors:** Mary Crea-Arsenio, Andrea Baumann, Jennifer Blythe

**Affiliations:** 1 62703 McMaster University, Hamilton, Ontario, Canada.

## Abstract

As part of its post COVID-19 recovery plan, the Canadian government is increasing the number of skilled immigrants, including Internationally Educated Nurses (IENs). However, pre-pandemic data show that IENs are underutilized and underemployed despite their education and experience. Focusing on the province of Ontario, this article explores trends in the IEN workforce and policies to address the nursing shortage. Barriers to IEN integration are reviewed and changes in the demographic and employment characteristics of IENs are analyzed. The disproportionate number of IENs employed in the Ontario long-term care sector, which has low wages and poor working conditions, emphasizes the need for policies that support the integration of IENs into the broader Canadian health system and increase their earning potential. To engage in strategic workforce planning and policy development, health leaders require access to nurse demographic and employment data that is timely and reflects the international and domestic labour supply.

## Introduction

Countries are contending with severe labour shortages as they struggle to recover from the impact of COVID-19.^1^ In Canada, the pandemic exacerbated vulnerabilities in the health system and deficits in the healthcare workforce.^2^ There are more than 28 million nurses worldwide, representing the greatest number of health human resources.^3^ However, at least 13 million more nurses will be needed over the next decade to fill labour shortages.^4^ The International Centre on Nurse Migration recommends that “the main source of new supply should be to invest in training more nurses domestically; a secondary source, open to countries that have the available resources, is to resort to active international recruitment.”^4^

As a top destination for newcomers, Canada admits Internationally Educated Nurses (IENs) through its various immigration streams.^5-7^ Yet despite their education and experience, their skills are often underutilized and many are underemployed.^7^ Immigrants who completed a bachelor's degree or higher in a professional nursing program outside of Canada were almost four times more likely to be overqualified (58%) in their employment than those who completed the same level of education in Canada (15%).^6^

As a requirement to practice, IENs must have their foreign credentials assessed against Canadian standards and approved by the relevant provincial or territorial regulatory college. This process is complex, lengthy and expensive and significantly contributes to the underutilization and underemployment of IENs.^7,8^ Accelerating their commensurate employment and workforce integration is paramount, particularly given Canada's ambitious immigration targets.^9^ Understanding how the IEN workforce has changed over time can help health leaders create strategies to improve IEN integration now and in the future.

Ontario is Canada’s most populous province and the most popular province for newcomers.^10^ However, it has a considerable shortage of nurses. The “average number of Registered Nurses (RNs) per 100,000 people is just 668”^11^ and there is a projected shortage of “33,000 nurses and personal support workers by 2028.”^12^ Using the Ontario case, this article examines trends in the IEN workforce and policy responses to address the nursing shortage. Literature on the barriers to IEN integration is reviewed and changes in the demographic and employment characteristics of IENs are analyzed. An overview of government initiatives created to transition IENs effectively and efficiently into the workforce is presented. The article concludes with a discussion of the issues highlighted by the trends, what the government is doing to address these issues and what more should be done.

## Barriers to IEN integration

In Canada, IENs make up 10% of the current nursing workforce.^13^ They are a valuable health human resource and can be used to offset shortages. However, they experience challenges with the licensure process and delays securing commensurate employment. A cross-sectional survey investigated how IENs manage the gap between their arrival in Canada and securing a job in their field. Respondents indicated using strategies such as working in nursing with a temporary licence, working in other health-related jobs and working in jobs outside the healthcare sector.^8^ During this time, some IENs experience downward occupational mobility and deskilling to the detriment of their professional trajectory and earning potential. As a result, many never qualify to practice as professional nurses.^7^

Internationally educated nurses come to Canada with nursing credentials and certification from their countries of origin but are not eligible to practice upon arrival. Their reinstatement as professional nurses is impeded by the time and resources required to become employment-ready.^13,14^ There is evidence of high levels of attrition at each stage of the IEN journey to commensurate employment.^15^ All IENs must first undergo evaluation and verification of their professional credentials by the National Nursing Assessment Service (NNAS) prior to registration with and licensure by the regulatory body in the province or territory in which they intend to practice.^16^

Ontario attracts the majority of IENs. Between 2016 and 2020, the number of IEN applicants to the College of Nurses of Ontario (CNO), the regulatory body for the province, more than doubled. In 2020, 37% of applicants to the CNO had received their nursing education outside of Canada.^17^ If an IEN’s education and practice do not meet the CNO’s requirements, he or she is directed to complete a bridging program to acquire the necessary nursing skills, knowledge, and judgement.^18^ Bridging programs may take a year or more to complete and are costly.

Financial constraints and temporal challenges, including the number of steps involved in the licensure process, have been linked to IENs selecting other career paths.^15^ The literature shows some IENs have been forced to take “survival jobs, working in positions that did not make use of their education.”^19^ One-quarter of nurse immigrants to Canada work in healthcare occupations outside nursing and approximately one-third work in low-skilled jobs outside healthcare.^6^

The final step in the registration process requires IENs to pass the NCLEX nurse examination. The cost to write the NCLEX-RN test in Ontario is $406.80 per attempt.^20^ The number of IENs writing the exam for the first time tripled from 669 in 2017 to 1,832 in 2021.^21^ On average, just over half of IENs pass the exam on their first attempt.^21^ Although there is no limit to the number of times eligible nurses can take the NCLEX, few make multiple attempts. As shown in Table 1, registration delays for IENs are greater than for Domestically Educated Nurses (DENs). Between 2016 and 2020, close to 90% of DENs achieved registration compared to 38% of IENs.

**Table 1.** Total number of College of Nurses of Ontario applicants achieving registration, 2016-2020.

Category	2016	2017	2018	2019	2020	Total
Ontario applicants	8,305	8,932	8,737	9,817	10,369	46,160
Became members	7,534	8,274	9,854	8,207	7,728	41,639
Internationally educated applicants^a^	2,967	3,758	4,710	4,556	6,315	22,306
Became members	1,263	1,335	1,883	1,958	2,123	8,562

^a^Does not include applicants from the United States.

Source: Office of the Fairness Commissioner.^17^

## The Ontario case

Ontario has a total population of 14.2 million and immigrants account for close to one-third of residents.^22^ Ontario Health oversees healthcare in six health regions across the province. There are 155 hospitals, 626 Long-Term Care (LTC) facilities and approximately 419 community health organizations (including home care).^14^ The nursing workforce includes RNs and Registered Practical Nurses (RPNs). The latter are referred to as Licenced Practical Nurses (LPNs) in all other provinces and territories. As requirements to practice, nurses in Ontario must register with the CNO and renew their membership annually. The CNO maintains a database of active nurses that includes demographic, education, and employment variables.

## Methodology

A secondary analysis of the CNO database was conducted to create a demographic and employment profile of IENs over a 10-year period (2012-2022). Descriptive statistics were calculated on age, employment status, sector of employment, work preferences, and actual work status. The analysis was conducted separately for RNs and RPNs. For each category of nurses, IENs were compared to DENs to examine between-group differences.

## Profiling the Ontario IEN workforce

In 2022, IENs accounted for 13% (21,605) of the total nursing workforce (169,340). The number and proportion of IENs registering with the CNO has increased over time. In 2022, 41% of new registrants were internationally educated; more than triple the number in 2020. With regard to nursing category, 60% of new IENs were registered as RPNs, while 40% were registered as RNs. Most IENs migrate from India and the Philippines, signalling a shift from traditional locations such as the United Kingdom.^23^ Over half settle in the Greater Toronto Area and large urban centres across the province. The majority are female and on average older than the domestic supply.

Internationally educated nurses are employed in hospitals, LTC facilities and community-based organizations. Table 2 shows the distribution of IENs and DENs by sector of employment.

**Table 2.** Number and percent of internationally educated nurses and domestically educated nurses by employment sector, 2022.

Sector	IENs		DENs		Total	
	#	%	#	%	#	%
Hospital	12,262	49	88,842	54	1,01,104	54
Community	3,918	16	36,338	22	40,256	21
LTC	6,943	28	22,136	14	29,079	15
Other	1,714	7	16,467	10	18,181	10
Not specified	10	.04	70	.04	80	.04
Total	24,847	100	1,63,853	100	1,88,700	100

Source: College of Nurses of Ontario registration database [unpublished data].

Although most nurses work in the hospital sector, twice as many IENs (28%) as DENs (14%) are employed in LTC. Overall, there are more RPNs (31%) than RNs (7%) in the LTC sector. However, 45% of the RPNs and 19% of the RNs employed in this sector are internationally educated.

**Figure 1.**
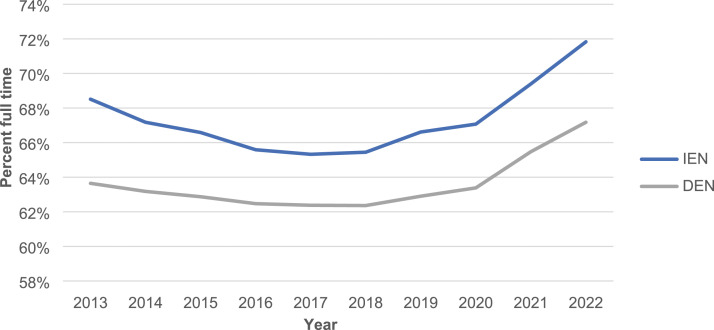
Percent of internationally educated nurses and domestically educated nurses in Ontario working full-time, 2013-2022.

Nurses are employed in Full-Time (FT), Part-Time (PT), or Casual (CAS) positions. Evidence demonstrates that maintaining a 70% full-time complement ensures continuity of care and stabilizes the nursing workforce.^24^ It is also important for nurse retention.^25^ The trend toward full-time employment of IENs has increased over time (Figure 1).

Internationally educated nurses have reached the 70% benchmark and a higher percentage of IENs than DENs work full-time.

### Preferred work status

Work preferences affect how well nurses integrate into employment. When preferences do not coincide with actual work, nurses experience less job satisfaction and increased intention to quit.^26^ According to CNO data, almost all nurses who are employed full-time prefer working in this capacity. Nurses employed part-time are less satisfied. In 2022, close to half of IENs and one-third of DENs employed part-time indicated a preference for full-time employment. Among nurses working part-time, preference for full-time employment varies according to nurse category. Regardless of immigrant status, proportionally more RPNs than RNs who work part-time prefer full-time status. This may be because RPNs have lower rates of full-time employment overall, leading to an increase in involuntary part-time work.

## Government initiatives

Several policy responses have been implemented to accelerate commensurate employment of IENs in Ontario. Pre-employment initiatives include the Ontario Bridge Training Program that offers immigrants “fast access to training and supports towards a licence. . . and assistance in finding employment in their profession.”^27^ Employment initiatives include the Nursing Graduate Guarantee, which provides incentives to healthcare employers to hire newly licenced IENs and support them via extended orientation and mentorship.^28^

Prompted by the nursing labour shortage additional initiatives have been introduced. The Supervised Practice Experience Partnership (SPEP) program helps eligible IENs complete the evidence to practice requirement for licensure and begin working as nurses in Ontario.^29^ The Enhanced Extern Program allows eligible employers to hire IENs into an unregulated role and work under the supervision of a nurse while completing registration requirements.^30^ There are also short-term initiatives such as provisionally subsidizing the cost of examination, application and registration for IENs (approximately $1,500), and allowing them to register in the temporary class.^31,32^

## Discussion and conclusion

Using the Ontario case, this article provides an analysis of the demographic and employment characteristics of IENs over a 10-year period to identify trends in and contributions of this workforce. The number of nursing vacancies in Ontario has increased substantially due to the stress of the pandemic, increased workloads and low domestic supply. In response, the provincial government implemented various initiatives to transition IENs effectively and efficiently into the workforce.

Findings reflect that the number of IENs entering the health system has been increasing over time with a concomitant decrease in the number of DENs. Between 2013 and 2022, the number of new IENs registering with the CNO increased from 13% to 24%, while the number of DENs decreased by 11%. The greatest growth occurred between 2020 and 2022 when the government increased investments intended to stabilize the nursing workforce in response to COVID-19. Although provincial initiatives have had some success in accelerating the integration of IENs, the most significant increase in numbers has been in the RPN category.

On average, IENs are older than DENs and have valuable and applicable healthcare experience from their home countries.^14^ India and the Philippines have become the main source countries for immigrant nurses. In terms of employment, there has been an increase in the percentage of IENs working full-time as compared to DENs. Although the percentage of full-time employment is high among IENs, findings from the current analysis demonstrate that involuntary part-time work is an issue. Almost half of IENs who were employed in part-time nursing positions in Ontario would prefer to work full-time.

Furthermore, data indicate that a greater percentage of IENs than DENs are employed in the LTC sector. This trend has been consistent over time for RNs and RPNs and predates the pandemic. Other Ontario case studies found that between 2011 and 2020, internationally educated RPNs “increased by 255%. . . with the majority working in LTC.”^23^ The LTC sector is characterized by lower wages, irregular working hours, chronic understaffing and poor work environments compared to the hospital sector.^26^ In addition, it has one of “the highest rates of workplace illness and injury”^33^ among staff. The disproportionate number of IENs working in LTC highlights their underutilization and emphasizes the need for policies that will enhance and accelerate their integration into the broader Canadian health system and increase their earning potential.^34,35^

Findings from this article have important implications for health leaders in Ontario and across Canada. Employing IENs has become a strategy to address the nursing shortage;^32^ thus, understanding the workforce profile of these nurses is necessary to ensure their efficient and effective transition into productive, commensurate employment. Even though IENs are an important source of supply and their numbers have increased over the last 10 years, they are still underrepresented and underutilized in the health system as compared to DENs. This is a critical issue because it prevents the creation of a health workforce that reflects the increasingly diverse population it serves.^14^ Moreover, it represents a significant loss of talent that prevents IENs from maximizing their earning potential.

To engage in strategic workforce planning, health leaders require access to nurse demographic and employment data that is timely and reflects the international and domestic labour supply. Having access to these types of data facilitates planning, strengthens recruitment and retention strategies, and ensures workforce continuity. It enables an organization to develop a plan that includes specific strategies, the means of achieving them and benchmarks against which expected outcomes can be measured. The successful government initiatives in Ontario could be replicated in other provinces and territories to improve IEN integration. Finally, preference for full-time work should be prioritized when creating strategies to retain nurses and stabilize the workforce.
